# Harnessing Genomics for Breeding *Lantana camara* L.: Genotyping and Ploidy Testing of Clonal Lines Through ddRADseq Applications

**DOI:** 10.3390/ijms26104898

**Published:** 2025-05-20

**Authors:** Angelo Betto, Fabio Palumbo, Damiano Riommi, Alessandro Vannozzi, Gianni Barcaccia

**Affiliations:** Department of Agronomy, Food, Natural Resources, Animals and Environment (DAFNAE), Campus of Agripolis, University of Padova, Viale dell’Università 16, 35020 Legnaro, PD, Italy; angelo.betto@phd.unipd.it (A.B.); damiano.riommi@unipd.it (D.R.); alessandro.vannozzi@unipd.it (A.V.); gianni.barcaccia@unipd.it (G.B.)

**Keywords:** ornamentals, plant breeding, genotyping by sequencing, genetic similarity, genetic structure, flow cytometry, chromosome count, ploidy prediction from SNP profiles

## Abstract

*Lantana camara* L. is sold worldwide for ornamental purposes, although it is also characterized by high invasiveness potential. Genetic and molecular data available for *L. camara* are still poor, and breeding is performed through conventional methods. This study focused on a molecular genotyping analysis through the ddRADseq method on an experimental collection of lantana clonal lines to evaluate the potential of molecular techniques in performing marker-assisted breeding, in favour of variety registration and in guaranteeing plant variety protection for the species. Although high genetic uniformity was observed in the population, a unique molecular profile was assigned to every line, indicating the effectiveness of the approach used. Interestingly, low degrees of heterozygosity were observed. In addition, the possibility of inferring ploidy levels through SNP profiles was assessed since it would avoid the necessity of previous biological knowledge and the use of fresh materials. Ploidy analysis is of high interest for lantana breeding to obtain less invasive triploids. Flow cytometry and chromosome counting were used for inference assessment. An nQuack framework provided correct results for the majority of the clonal lines, confirming its effectiveness. These findings encourage the adoption of molecular systems to help breed minor species such as *L. camara.*

## 1. Introduction

*Lantana camara* L. is a vigorous shrub species belonging to the Verbenaceae family that originated in South America and Central America and became naturalized after its import to North America, Australia, India and Africa [1,2,3]. Lantana, which is sold worldwide for ornamental purposes due to the attractiveness of its flowers and leaves, is notable for its use in traditional medicine [4], antimicrobial activity [5], antioxidant potential against free radical-associated diseases [6,7], and especially invasiveness in many countries [8,9,10,11]. In fact, it is classified as one of the 100 most invasive plants in the world [12], and in Florida, the survival of the native plant *Lantana depressa* Small is endangered, as these two species are interfertile [13,14,15]. *L. camara* (2n = 2x = 22) has been reported to be self-compatible and capable of both self- and cross-pollination, with thrips as the main pollinators, although there is no agreement in the scientific literature on the frequency of self-pollination [16,17,18,19]. Lantana natural populations and commercial lines can be autopolyploid or allopolyploid, particularly tetraploids and hexaploids [20,21]. Interestingly, lantana is capable of producing unreduced female gametes, hence increasing the ploidy level, which is believed to be the reason why some triploids are fertile [22,23]. The molecular data available for *Lantana camara* are very limited: a reference genome was only released in 2024 [24], and few transcriptomic studies have been carried out mainly to investigate traits of economic interest, such as flower colour, gamete formation and phenylpropanoid biosynthesis [25,26,27].

Although no study to date has specifically focused on the total genetic diversity within the species, the inter-varietal variability is theoretically expected to be very high, considering the high number of cultivars present in the market and the notable genetic structure found in several invasive lines, which also revealed to be linked to important traits like flower colour [19]. However, *L. camara* commercial lines, as those of the majority of ornamentals, are primary propagated clonally through cuttings. Therefore, ornamental varieties often show very low intra-varietal variability, primarily resulting from somaclonal variations and environmental factors. Moreover, it is not uncommon for new varieties to be developed from pre-existing ones, as is the case with essentially derived varieties (EDVs), which can be distinguished from their relative initial varieties (IVs), even for a single trait. In the latter case, it is possible that inter-varietal variability between EDV and IV is very low too [28].

Plant variety protection (PVP) through its inclusion in national and international official registers allows the identification of plant material of unknown origin, avoiding fraud and providing a significant contribution to guarantee plant breeder rights (PBRs) [29]. Considering that this species is vegetatively propagated, embezzlement of materials developed by others is theoretically easily achievable. The International Union for the Protection of New Varieties of Plants (UPOV) and the European Community Plant Variety Office (CPVO) provide guidelines for variety registration that rely on Distinctiveness Uniformity and Stability (DUS) tests, involving morphological and physiological descriptors [30,31,32,33]. To date, technical protocols directly developed by CPVO and UPOV are not available for lantana, but the guidelines created by the German Bundessortenamt have received approval from CPVO to be exploited by the European authority itself [34]. They comprehend 22 descriptors, including leaf blade width, flower bud colour, and plant height, each to be recorded on the basis of discrete evaluation classes [35]. The use of morphological descriptors could be considered sufficient to identify varieties of exclusively ornamental species without the need for additional tests evaluating productivity parameters, such as Value for Cultivation and Use (VCU) [36]. However, for ornamentals, the need to produce varieties that are sustainable, resilient and able to provide ecosystem services is increasing [37], and these traits cannot be evaluated through morphological parameters. The molecular characterization of varieties and breeding lines can potentially be useful for this purpose, in addition to (i) ensuring compliance with PBRs, (e.g., by detecting unknown plant materials); (ii) supporting breeding processes (i.e., marker-assisted breeding, MAB), e.g., by optimizing crosses and reducing the number of breeding lines to be maintained and (iii) accelerating the selection of agronomically important traits through assays (i.e., marker assisted selection (MAS)) [38,39,40,41]. Several types of molecular markers, including simple sequence repeats (SSRs) and single nucleotide polymorphisms (SNPs), and several approaches, such as double digestion restricted site-associated DNA sequencing (ddRADseq), have been successfully exploited for the genotyping of a wide range of plant species [42,43,44,45,46]. According to the CPVO protocols, the molecular characterization of a variety during its registration is possible but not mandatory [47].

In addition to variety selection and registration, molecular genotyping can provide great assistance in estimating ploidy. Determining the ploidy level of a plant is crucial from a breeding perspective since it can affect specific traits of interest and their segregation patterns [48,49,50,51]. In *L. camara*, tetraploids are characterized by greater aerial vigour and growth habit asymmetries, whereas the development of triploid varieties is highly important for reducing invasiveness. Indeed, triploids demonstrate less fertility than individuals with other ploidy levels do [13,21,22]. Ploidy is usually estimated through chromosome count or flow cytometry (FCM) [52,53], but both techniques require fresh tissues and in-depth knowledge about chromosome number and genome size, which are not always available or are difficult to manage [54]. The possibility of exploiting molecular data to determine ploidy would help overcome many of these obstacles. On this matter, bioinformatic tools relying on high-throughput sequencing (HTS) data and exploiting allelic ratios present at each heterozygous position have been developed [54,55,56,57].

In our study, we conducted a de novo ddRADseq-based genotyping analysis useful for MAB applications aimed at variety selection, and for registration and protection of already developed plant materials in lantana. By examining a breeding population of clonal lines, we aimed to determine whether the DNA sequencing system could uniquely genotype individual plant lines and accurately determine their genetic relationships, even in cases of low genetic diversity and limited genealogical data. In addition, we utilized molecular profiles to infer the ploidy levels of a subset of lines, which had previously been assessed through chromosome counting and FCM. This approach allowed us to evaluate the effectiveness of the limited portions of the genome sequenced in predicting ploidy levels for in-depth genotyped clonal lines.

## 2. Results

### 2.1. Genetic Similarity Estimates and Genetic Structure Analysis of the Breeding Population

A de novo ddRADseq analysis was carried out on 96 lantana samples from a core collection of 93 *L. camara* (identified as LaCaBL#) and 3 *L. montevidensis* (identified as LaMoBL#) breeding clonal lines. Sequencing produced a total of 989,816,600 demultiplexed reads, with an average of 10.3 M reads per sample and a mean read depth (MR) per sample of 10.8. A total of 16,275 consensus loci were identified, 13,781 of which were polymorphic. An initial number of 61,005 polymorphic sites was retrieved after variant calling, which was reduced to 14,039, with the filtering of positions showing the presence of missing values higher than 10% of the samples.

The genetic similarity (GS) analysis produced unique molecular profiles for all the samples considered, and no pairwise comparison resulted in genetic similarity estimates of 100%. The GS values ranged from 45.2% (LaCaBL34-1 vs. LaMoBL-02) to 96.2% (LaCaBL09-2 vs. LaCaBL09-4), with an average of 84.2 ± 6.5% (Table S1). The number of discriminative polymorphic loci between the two most similar breeding lines (BLs) was 533, whereas the calculation between the two most genetically different BLs led to a value of 7693. The *L. montevidensis* samples were the lines with the lowest average GS in relation to the other BLs. In Table S1, the average number of observed alleles (Na) per locus, observed heterozygosity (Ho) and percentage of private alleles (PAs), calculated per BL, are also reported. Ho values ranged between 5.2% and 29.2%, with an average of 15.6 ± 4.8%, highlighting a high level of homozygosity for all BLs. Four out of the five tetraploids presented some of the highest Ho values. LaMoBL03 had the highest PA% value of the collection (40.8%).

**Figure 1:**
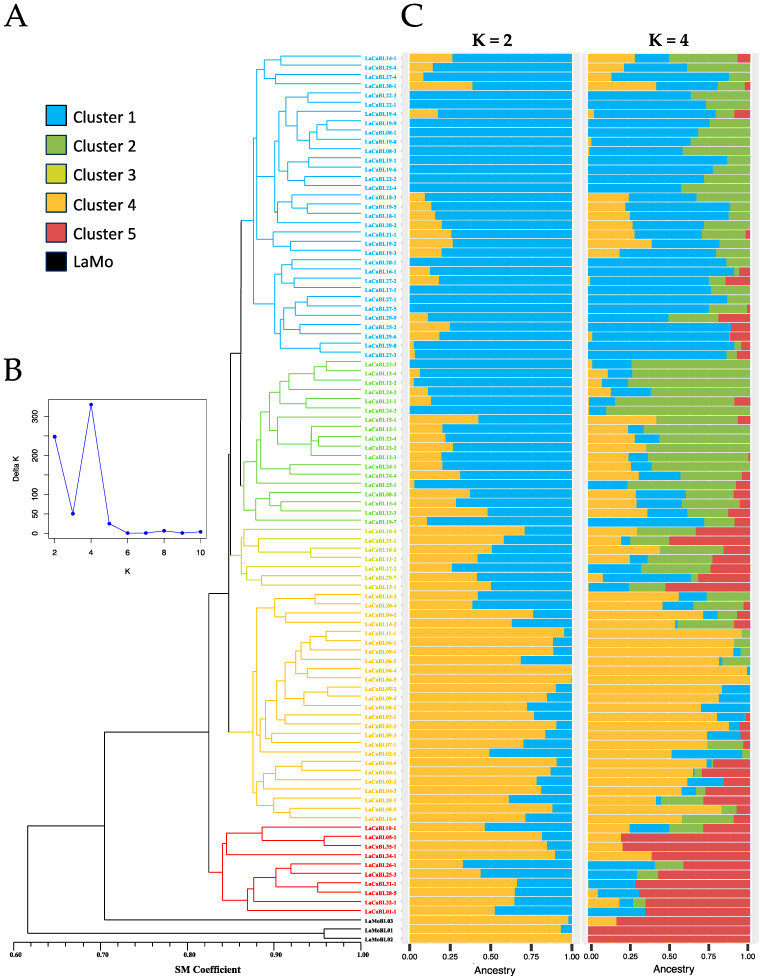
Genetic similarity (GS) and genetic structure analysis results based on the molecular profiles of 93 *L. camara* (LaCaBL#) and *3 L. montevidensis* (LaMoBL#) clonal lines obtained with 14,039 SNPs via a ddRADseq approach. (**A**). Unweighted pair group method with arithmetic mean (UPGMA) dendrogram based on GS analysis performed with the simple matching (SM) coefficient. Different colours represent the highlighted clusters. (**B**). Genetic structure putative ancestor number (K) likelihood (**C**). Graphic representation of the genetic structure analysis considering K = 2 and K = 4. Different colours represent different putative ancestors.

An unweighted pair group method with arithmetic mean (UPGMA) dendrogram based on the GS analysis highlighted five different clusters (Figure 1A).

As expected, the three *Lantana montevidensis* samples clustered apart from the *L. camara* accessions. Interestingly, the five *L. camara* lines whose tetraploidy was assessed through FCM (i.e., LaCaBL05-1, LaCaBL31-1, LaCaBL32-1, LaCaBL34-1 and LaCaBL35-1) grouped together within the same cluster (Cluster 5). The same cluster was also the most isolated of the *L. camara* group. The genetic structure analysis revealed the highest values for K = 2 (Delta K = 248.1) and K = 4 (Delta K = 330.5) (Figure 1B). The results were considerably in agreement with the UPGMA clustering: for K = 2, Cluster 1 and Cluster 2 shared a common origin, unlike Cluster 4, Cluster 5 and the *L. montevidensis* group. Considering K = 4, Clusters 1, 2 and 4 were all assigned to as many ancestors, whereas the Cluster 5 and *L. montevidensis* groups clustered together, as already observed for K = 2. Cluster 3 was admixed mainly for both K = 2 and K = 4 (Figure 1C). Simple matching (SM) coefficient-based GS analysis was also carried out considering clusters obtained with UPGMA grouping. Compared with all the other clusters, the *L. montevidensis* group presented a lower GS, with an average of 58.6 ± 2.9%, with an overall GS in the *L. camara* group (Clusters 1–4) of 88.6 ± 2.9%. *L. montevidensis* also presented the highest mean value of Ho (28.3%). The same value was lower in the *L. camara* group, ranging from 13.35% in Cluster 3 to 19.9% in Cluster 4 (Table 1).

Genetic statistics were also computed considering clusters, including PA percentage, polymorphic locus percentage (PL%), Ho, expected heterozygosity (He) and the inbreeding coefficient (Fis). Fis was slightly positive for all clusters, with the sole exception of Cluster 4, highlighting a general deficit of heterozygosity. The statistical measurements were relatively similar for all the clusters, with the exception of the *L. montevidensis* group, for which consistently higher values were observed, except for the PL % (Table 2).

**Figure 2:**
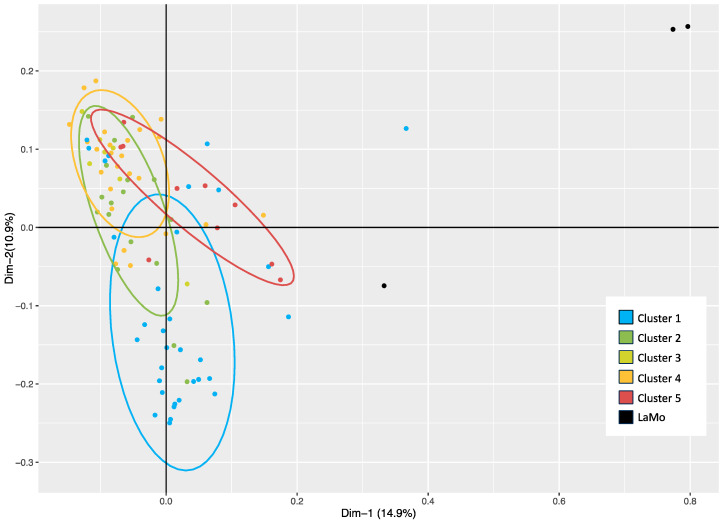
Principal coordinate analysis (PCoA) centroid based on genetic similarity (GS) analysis of the molecular profiles of 93 *L. camara* and 3 *L. montevidensis* clonal lines obtained from 14,039 SNPs via the ddRADseq approach. Coordinates explain the highest genetic variability (values reported: Dim%), and ellipses represent the clusters highlighted with an unweighted pair group method with arithmetic mean (UPGMA) clustering.

A principal coordinate analysis (PCoA) explained a low percentage of the total observed variance, equal to 25.8%. The *L. montevidensis* lines were clearly separated from the remaining samples, but the *L. camara* clusters overlapped and were therefore not distinguishable (Figure 2).

### 2.2. Ploidy Assessment by Flow Cytometry

**Figure 3:**
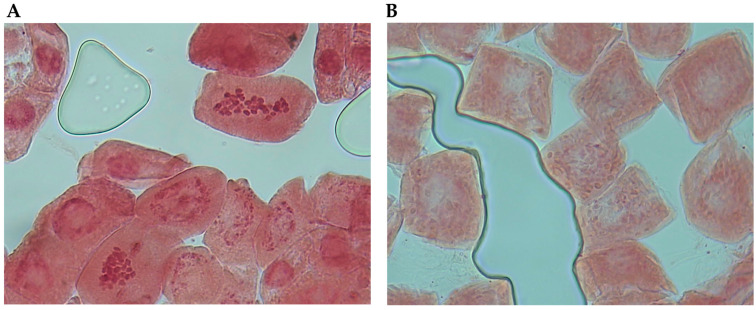
Chromosomes in metaphase stained with fuchsine at 100× magnification from the roots of the *L. camara* L. (**A**). Triploid line LaCaBL33-1 and (**B**). Tetraploid line LaCaBL32-1.

FCM analysis was carried out to determine lantana sample ploidy classes. From previous tests on the population genotyped with ddRADseq, a putative triploid (LaCaBL33-1) and a putative tetraploid (LaCaBL32-1) were selected for use as internal references. To assess their ploidy class, the two BLs were subjected to a chromosome count and a genome size analysis. The chromosome count was possible on a minimum of four clearly observable metaphase plates in both lines and revealed 32.5 ± 5.3 and 44.8 ± 2.9 chromosomes per cell for LaCaBL33-1 and LaCaBL32-1, respectively (Figure 3).

**Figure 4:**
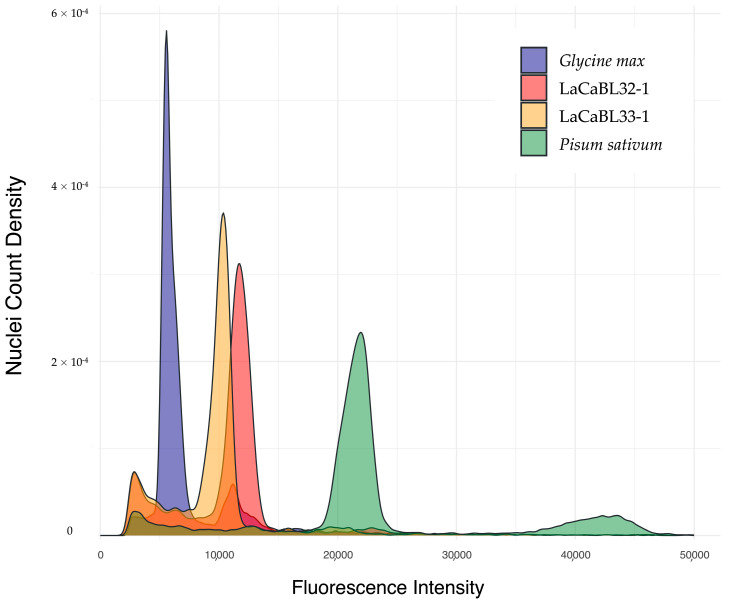
Overlay histogram of the fluorescence intensity in relation to the nuclei count kernel density estimation relative to a flow cytometry analysis for the genome size estimation of *Lantana camara* L. triploid (LaCaBL33-1) and tetraploid (LaCaBL32-1) breeding clonal lines, *Glycine max* Merr. cv. “Polanka” (2C DNA content: 2.50 pg) and *Pisum sativum* L. cv. “Ctirad” (2C DNA content: 9.09 pg) [58].

The determination of the 2C DNA content was carried out with FCM through comparison of the fluorescence intensity of selected lines to that of standard references in co-chopping processing. The standard references for other species were accessions with known genomic dimensions, particularly *Glycine max* Merr. cv. “Polanka” and *Pisum sativum* L. cv. “Ctirad”. The results revealed average 2C DNA contents of 4.51 pg (4.41 × 10^3^ Mb) and 6.09 pg (5.96 × 10^3^ Mb) for LaCaBL33-1 and LaCaBL32-1, respectively (Figure 4; Table 3).

Through this process, LaCaBL33-1 and LaCaBL32-1 were confirmed to be triploid and tetraploid, respectively.

LaCaBL32-1 was used as a control line in a co-chopping FCM analysis for ploidy determination of random lantana BLs from the collection genotyped through the ddRADseq approach. The peak quality observed in the FCM analysis was generally high, with a coefficient of variation (CV) in every case lower than 8.0%. Different replicates of the same sample and different samples within the same range presented very similar fluorescence intensity deltas between the control and sample values. Considering the tetraploid control, the control/sample ratios obtained were very close to 2.0 for the first range and 1.3 for the second. For the third range, a single peak was observed, which indicates the coincidence of the sample and the control signal (Table 4).

The first value corresponds to the tetraploid-diploid genome size ratio (4:2), and the second corresponds to the tetraploid-triploid genome size ratio (4:3). Among the 13 analysed BLs excluding the control lines, six and three presented the first and second ratios, respectively, and hence were diploid and triploid, whereas four BLs presented a single signal and therefore were demonstrated to be tetraploid.

### 2.3. Ploidy Prediction Using ddRADseq Data

**Figure 5:**
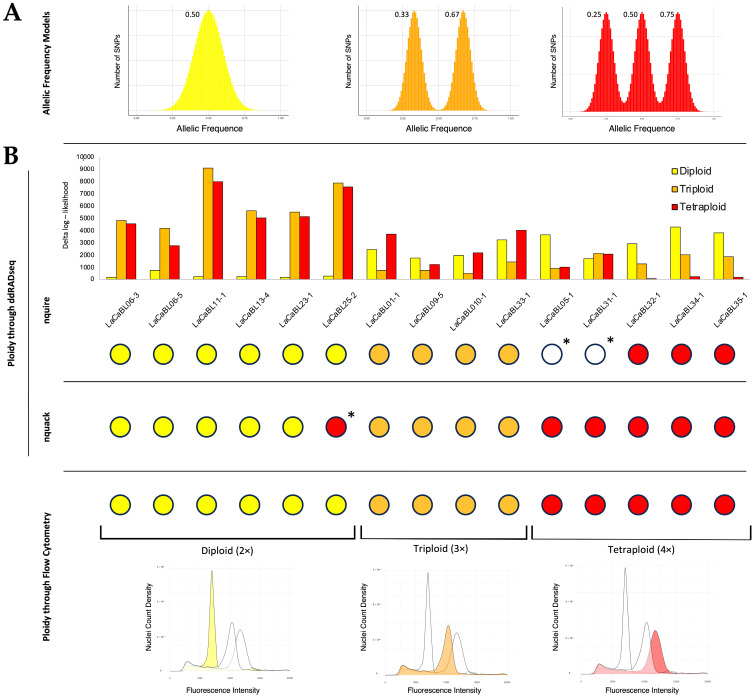
(**A**). Models of SNP distribution per allelic frequency for diploid, triploid and tetraploid organisms. (**B**). Likelihood of deviation from the models of the SNP distributions retrieved through the nQuire framework and ploidy predictions obtained through the nQuack framework from ddRADseq SNP profiles of 15 *L. camara* L. clonal lines in comparison with ploidy data derived from flow cytometry. *: lines for which the prediction did not match the flow cytometry results.

ddRADseq data produced for the 15 lantana BLs also analysed through FCM, comprehending the two controls (LaCaBL33-1 and LaCaBL32-1), were further investigated to infer ploidy levels on a molecular basis. For each line, the number of SNPs per allelic frequency considering heterozygous loci was taken into account by exploiting the nQuire framework. These data were compared to the model for every expected ploidy class (diploid, triploid and tetraploid) (Figure 5A), and the minimal deviation from the model was calculated, which represented the most likely ploidy level.

The nQuire analysis resulted in the assignment of a specific ploidy class for 13 out of 15 BLs. The likelihoods of the remaining two lines (LaCaBL05-1 and LaCaBL31-1) did not significantly differ among the different models. With respect to lines for which a unique level was provided by the prediction framework, all assignments were in agreement with those derived from FCM (Figure 5B). The likelihood differences between the different models for every sample were very high, with the only exception being LaCaBL09-5. For this BL, the likelihood for the hypothesized class, triploidy, was slightly different from that for the other two classes. The tested approach using nQuire was able to assign the right ploidy level to all six diploids and four triploids, whereas only three out of five tetraploids were correctly identified. In addition, alternative ploidy prediction through the nQuack framework, which exploits a similar estimation approach but with specific SNP filtering, was performed. Unlike the nQuire estimation, the tool returned very high likelihoods towards a specific ploidy level for every sample, reaching 100% in the majority of cases; hence, no undefined results were obtained. Every prediction was in agreement with the FCM results, except for LaCaBL25-2, to which tetraploidy was assigned instead of diploidy (Figure 5B). Therefore, the nQuack approach was able to assign the correct ploidy class to every triploid and tetraploid and to five out of the six diploids tested.

## 3. Discussion

In this study, we performed ddRADseq analysis on 96 lantana experimental clonal lines to assess the effectiveness of this genotyping-by-sequencing (GBS) technique in characterizing plant accessions with low genetic diversity and unknown ancestry. Overall, this approach produced unique molecular profiles for every BL, although a high level of genetic uniformity was detected within the collection. Additionally, it allowed the retrieval of several pieces of information about the collection of interest, including the GS between different lines, heterozygosity degree, and PA presence, which are parameters of great interest for evaluating the breeding work carried out. Eventually, ddRADseq achieved clustering of samples, which can be helpful for MAB, e.g., allowing us to find the most genetically distant BLs to cross and hence maximize the observed variability in the progeny [38]. Moreover, this approach can be useful for favouring variety registration, not only guaranteeing PVP but also simplifying DUS tests. In fact, the number of reference varieties used to assess candidate distinctiveness can be reduced to only the most genetically similar ones [47].

The fact that the genotyping analysis was able to distinguish highly related breeding lines suggests that it can also be exploited for the identification of EDVs. This finding can support the claim that IV breeders demand royalties from EDV sales on the basis of their predominant work [59]. In addition, whether the phenotypic differences observed in the comparison of a candidate with already registered varieties refer to traits of interest involved in nonmorphological aspects (e.g., related to sustainability or ecosystem services), traditional DUS tests are not sufficient. Instead, assessing distinctiveness through molecular diversity may be useful in these cases, extending to ornamental species the proposal of Gilliland et al. [60] for herbages, named value-molecular DUS (vmDUS) testing.

The effectiveness of ddRADseq as a relatively low-cost genotyping technique [61,62] is in agreement with studies of various species of agronomic interest for the analysis of breeding populations [38,39,40,44,63,64]. The de novo approach was chosen since accessions of two species were exploited for the analyses, and the use of an *L. camara* genome as a backbone for the read mapping could have led to an important loss of loci for *L. montevidensis* lines [65,66,67,68]. In addition, assessing the effectiveness of de novo approaches to reduced representation library (RRL) sequencing can be useful for minor species lacking reference genomes, as the majority of ornamental plants. Indeed, in those cases there might not be economic convenience in applying deeper or more expensive analyses for genotyping purposes [69,70,71]. Considering lantana, ddRADseq was previously used only for genetic structure analysis of invasive populations in India [19]. Another GBS technique, namely diversity array technology sequencing (DArTseq), was exploited for the same purposes on some Australian biotypes [72]. Other molecular genotyping studies have been performed for ecological and taxonomic purposes on wild lantana populations with other molecular marker typologies: microsatellites and chloroplast spacers [73,74], intersimple sequence repeats (ISSRs) [15,75], and DNA barcoding and restriction fragment length polymorphisms (RFLPs) [15]. However, this was the first attempt to apply GBS to experimental lantana BLs to distinguish very similar genotypes.

With respect to the lantana collection analysed, a correlation between clustering and ancestry likelihood results was found, strengthening the genotyping analysis reliability. Interestingly, high homozygosity levels were found for the BLs. This finding was surprising for two main reasons: lantana is considered to be facultatively allogamous, and BLs are clonally propagated; thus, high homozygosity levels are not needed for trait stability and purity maintenance [14,16,76]. High homozygosity can be explained by self-pollination as a prevalent reproductive system, or, by contrast, through cross-pollination occurring among highly genetically similar lines. If we consider the first scenario, our results would be consistent with the observations made by Praveen et al. in the study of some invasive biotypes of *L. camara* in India. Also in this case, the authors identified a low percentage of heterozygous loci, suggesting the possibility that, in specific contexts, the species may predominantly reproduce through self-pollination [19]. The author advanced the hypothesis that, while cross-pollination seems to characterize the species in native contexts, a shift from cross to self-fertilization could occur when *L. camara* is introduced in new environments, as already observed in other species [19,77,78]. This observation would also support the hypothesis that *L. camara* is a single clearly defined species rather than a species complex derived from interspecific hybridization events. [15,73,79,80]. On the other hand, if we consider the hypothesis that *L. camara* is indeed a species that predominantly reproduces through cross-pollination to be valid, it is plausible that the high level of homozygosity observed in the BL population is the result of crosses between accessions with a high degree of genetic similarity. The levels of genetic diversity within the population were found to be particularly low for some clusters, to the point that some BL clusters were indistinguishable in the PCoA analysis. An increase in the overall variability through the introduction of external genetic sources into the breeding program is needed for this population whether the goal is to achieve innovative combinations of traits and develop new varieties. As expected, *L. montevidensis* BLs, which were included in the analysis as benchmarks, were highly distinct from all the other lines in every analysis performed. Two of the three BLs, LaMoBL1 and LaMoBL2, were similar, whereas LaMoBL3 was genetically different, with a high percentage of PAs and the greatest degree of heterozygosity. Studies focused on bigger *L. montevidensis* collections are needed to further investigate the genetic variability observed among the accessions of this species. According to literature, the two species seem not to be highly related considering the genetic variability of the entire genus, although taxonomy has not been established with certainty [1]. Both are capable of producing fertile interspecific hybrids with more related species [1,15,80,81], and *L. camara* × *L. montevidensis* hybrids have been artificially obtained for horticulture purposes [82]. The fact that *L. montevidensis* BLs clustered separately from the other samples might be a clue to the correctness of the obtained grouping.

*Lantana* is a genus for which unreduced gamete occurrence is not rare, which may facilitate polyploidization and interspecific hybridization [14,15]. In addition, plants of this genus were found to produce unreduced female gametes, which may explain the triploid occurrence [23,76]. Ploidy determination is highly important in breeding programs, especially in lantana, for which the development of infertile triploid varieties or interspecific hybrids to counteract invasiveness is a challenge [13,22]. Ploidy assessment requires specific analyses and fresh material availability [52,53,54]. To assess whether ploidy can be estimated by taking advantage of next-generation sequencing (NGS) data, we compared the ploidy estimates retrieved with FCM with those obtained via ddRADseq. In the FCM analysis, a lantana control accession of known ploidy was exploited to confirm the trait level in other lines. For the development of the control line, the ploidy level of a putative triploid and tetraploid was confirmed with genome size analysis through FCM and chromosome counting. In particular, the calculated genome sizes were 4.51 pg and 6.09 pg for the putative triploid and tetraploid lines, respectively. The average value obtained in the literature for the 2C DNA content of *L. camara* was approximately 3.0 pg [83,84]. The ratios between the data obtained and the literature information were therefore 3:2 and 2:1, corresponding to those characterizing triploids and tetraploids, respectively. In addition, the chromosome counts of metaphase cells from the two putative references revealed 33 and 44 chromosomes, respectively, which are still equal to the values reported for triploids and tetraploids [24,76,85]. These observations confirmed their ploidy level and allowed them to be used as a control for screening 13 lantana BLs via FCM. The analysis revealed six diploids, three triploids, and four tetraploids. The control line development and the production of a rapid assay for screening large breeding populations for ploidy are tools of great interest for breeding, especially in the ornamental sector.

The ploidy levels of the 15 BLs subjected to FCM were predicted through nQuire and nQuack statistical frameworks [56,57] from the sequences obtained with ddRADseq analysis on the basis of allele frequencies. The nQuire approach was able to detect the correct ploidy of 13 out of 15 lines, and the two samples for which the analysis yielded undefined results were assessed as tetraploid through FCM. Therefore, the tool was proven to yield the correct results for all the diploids and triploids tested, but it cannot be considered effective for tetraploids. Polyploids may be more difficult to detect owing to the greater genetic complexities they present and the greater number of allelic copies per locus, which might more likely lead to allelic frequency alterations from ploidy class models. The fact that uncertain outputs were provided for the two BLs for which prediction failed, instead of a high likelihood towards a unique but wrong ploidy level, may enhance analysis reliability in the case of certain results. However, this is not sufficient to consider nQuire a promising tool for lantana ploidy estimation. Conversely, nQuack analysis was able to assign the correct ploidy class to all tetraploids, in addition to triploids and five out of the six diploids. Therefore, in this study, nQuack proved to be effective overall in ploidy estimation for all tested classes. The better prediction performance in comparison to nQuire may be due to the greater number of options available for filtering the SNP distributions, as claimed by the framework developers [56]. Importantly, the tested approach also resulted in promising outcomes considering the relatively low genome coverage and sequencing depth characterizing ddRADseq [86,87]. Several studies have exploited nQuire or nQuack for ploidy estimation in various species, but only a few have evaluated their efficacy by comparing the results with those obtained via FCM [56,88]. Despite these promising results, further analyses in different populations are necessary to evaluate the performance of the tool for estimating ploidy levels in *L. camara.*

## 4. Materials and Methods

### 4.1. Plant Materials

Leaf samples from 96 lantana breeding clonal lines (BLs), specifically 93 *L. camara* and 3 *L. montevidensis* BLs used as benchmarks, were provided by Gruppo Padana S.S. (Paese, TV, Italy), a nursery company specialized in the production, selection and collection of young plants from seeds and cuttings. The analysed population represented the total nursery company collection. Genomic DNA (gDNA) was extracted from 100 mg of leaf tissue via a DNeasy Plant Mini Kit (Qiagen, Valencia, CA, USA) following the manufacturer’s instructions. gDNA quality and concentration were assessed via a NanoDrop 2000c UV—Vis spectrophotometer (Thermo Fisher Scientific Inc., Pittsburgh, PA, USA) and agarose gel electrophoresis (1% agarose/1 × TAE/1 × SybrSafe DNA stain (Life Technologies, Carlsbad, CA, USA)).

### 4.2. ddRADseq Library Preparation

ddRADseq libraries were produced following an IGA Technology Services (Udine, Italy) custom protocol developed by Peterson et al. [61], with some modifications. Specifically, gDNA was fluorometrically quantified and normalized to a uniform concentration, and 300 ng was double digested with 2.4 U of both SphI and EcoRI endonucleases (New England BioLabs, Ipswich, MA, USA) in a 30 μL reaction supplemented with CutSmart Buffer. Differently from the original protocol, the digestion incubation was carried out at 37 °C for 90 min and then at 65 °C for 20 min. Digested DNA is subsequently ligated with 160 U of T4 DNA ligase (New England BioLabs) to 2.5 pmol of overhang barcoded adapters for both cut sites in 50 μL reactions incubated at room temperature for 120 min and, followed by 65 °C for 20 min. Digested DNA from 48 samples was pooled collecting 5 μL of DNA per sample and the same operation was carried out for the remaining 48 samples. The two pools were purified with 1.5 volumes of Agencourt AMPureXP beads (Beckman Coulter, Indianapolis, IN, USA). The fragments included in the range 400 bp–600 bp were collected on a BluePippin instrument (Sage Science, Beverly, MA, USA). The eluted fraction was amplified with indexed primers using Phusion High-Fidelity PCR Master Mix (New England BioLabs) of 50 μL with the following thermal profile: one cycle of 95 °C for 3 min, 10 cycles consisting of 95 °C for 30 s, 60 °C for 30 s, and 72 °C for 45 s, and a final cycle at 72 °C for 2 min. The products were purified with 1 volume of Agencourt AMPureXP beads. The resulting libraries were checked with both a Qubit 2.0 fluorometer (Invitrogen, Carlsbad, CA, USA) and a Bioanalyzer DNA assay (Agilent Technologies, Santa Clara, CA, USA). Libraries were normalized to 200 pM and sequenced in two runs, one per pool. Sequencing was carried out with 150 cycles in paired-end mode on a NovaSeq 6000 instrument following the manufacturer’s instructions (Illumina, San Diego, CA, USA).

### 4.3. ddRADseq Data Analysis for Assessing Genetic Diversity in Lantana Clonal Lines

Read processing was carried out via a de novo approach through Stacks v2.61 software [89]. In particular, raw reads were demultiplexed according to the restriction enzyme recognition motif and trimmed at the 3′-end to obtain a minimum average Phred quality score of 20 over a window of ten bases. Moreover, sequences (i) containing undetermined bases; (ii) having a final length of less than 64 bases; and/or (iii) presenting 5′-ends that did not match the restriction enzyme site were filtered out. Through the core modules ustacks, cstacks and sstacks with default parameters, the processed reads were assembled per sample into exactly matching stacks, a set of de novo loci across the population (catalogue) was created, and sample stacks were matched against the catalogue, allowing up to 5% difference. SNP calling was performed through gstacks command with these parameters: marukihigh model, SNP discovery alpha threshold of 0.10, genotype call alpha threshold of 0.10. The obtained variants were filtered using the populations command, setting the option –R = 0.80 in order to retain only loci that are represented in at least the 80% of the whole metapopulation and with cutoff --max-obs-het = 0.8 in order to process a nucleotide site at a locus with an observed heterozygosity at maximum of 80%. Further filtering was carried out with the missingno function of poppr v2.9.6 package of the RStudio software (Posit, Boston, MA, USA), in order to retain variants with maximum missing data of 10%.

The obtained SNP profiles were used to estimate the GS in all pairwise comparisons on the basis of the SM coefficient exploiting the NTSys software (NTSYSpc 2.21w) [90]. The latter was used also for the creation of an unweighted pair group method with a UPGMA dendrogram starting from the GS analysis data. A PCoA was carried out through the dudi.pco function of the RStudio ade4 package [91]. The average number of observed (Na) and effective (Ne) alleles per locus, the observed (Ho) and expected (He) heterozygosity, and the percentages of polymorphic loci (PLs) and private alleles (PAs) per individual and per cluster highlighted by UPGMA were calculated either with hierfstat package (0.5-11) [92] basic.stat function or with a manually developed R script. In addition, a genetic structure analysis with STRUCTURE v.2.2 software [93], which exploits a Bayesian clustering algorithm, was carried out. The following parameters were used: founding groups ranging from 1 to 10, 10 replicate simulations per K, burn-in of 20,000, and a final run of 100,000 Markov chain Monte Carlo (MCMC) steps. The K values characterized by the highest likelihood were selected using Structure Harvester software (0.6.94) [94].

### 4.4. Flow Cytometry, Chromosome Counting and ddRADseq Data Analysis for Ploidy Assessment

Ploidy analysis of lantana samples was carried out through FCM, and the cellular nuclei were extracted in three biological replicates per BL consisting of different leaves, following the CyStain PI Absolute P protocol (Sysmex Partec, Görlitz, Germany). In particular, approximately 100 mg of fresh leaf tissue was treated with 500 μL of Nuclei Extraction Buffer, ground, and filtered with 30 μm CellTrics filters, with the subsequent addition of 2 mL of Staining solution (1982 μL of Staining Buffer, 12 μL of Propidium Iodide, and 6 μL of RNAse A 3.3 ng μL^−1^) (Sysmex Partec). Sample analysis was carried out through a CyFlow Ploidy Analyser (Sysmex Partec) flow cytometer with the following parameters: green laser Nd-YAG wavelength, 532 nm; power, 30 mW; and flow, 4 μL s^−1^. The fluorescence intensity data were analysed via Flowing Software 2 (Turku Bioscience, Turku, Finland), and flow histograms were created with RStudio. Ploidy classes were determined through simultaneous processing of each single sample with a lantana control line (co-chopping) and calculation of the fluorescence intensity ratio between the sample and reference. The ploidy level of the lantana control line was determined through genome size analysis and chromosome count. Genome size was estimated following the same FCM protocol, co-chopping samples and reference standards belonging to other species, with known 2C genome sizes. Standards containing *G. max* cv. “Polanka” (2C DNA content: 2.50 pg) and *P. sativum* cv. “Ctirad” (2C DNA content: 9.09 pg) were kindly provided by Prof. Doležel [58]. DNA content values were calculated by comparing the sample and standard fluorescence intensities at the G0/G1 peak.

Chromosome counting was carried out by treating fresh root tips as described in the Feulgen staining procedure [95]. In particular, the following protocol was used: pretreatment with 0.4% colchicine for 3 h, fixation in Carnoy solution for 1 h, hydrolysis in 1 N HCl at 60 °C for 8 min, and staining with leuco–basic fuchsine for 2 h. Subsequently, the root tips were squashed in an aceto-orcein solution on microscope slides, and chromosomes were observed with a Leitz Diaplan optical microscope (Leica Microsystems, Wetzlar, Germany) at 100× magnification. Images were taken with Leica LAS–EZ 3.0 imaging software (Leica Microsystems) changing the microscope focus for chromosome counts in different space levels. At least four clearly observable metaphase plates per BL were counted.

Ploidy estimation through ddRADseq-derived molecular profiles was carried out via the nQuire [57] and nQuack [56] statistical frameworks. The frameworks exploit the binary aligned map (BAM) files created for ddRADseq read processing to measure the number of SNPs per allelic frequency at all heterologous sites of each BL. For nQuire, a minimal read sequencing depth for reads of 30× and the automatic denoise command were set, while nQuack was allowed to add, in addition to the same minimal depth and a different model for the denoise command, a truncation of data relative to allelic frequencies inferior to 0.15 and superior to 0.85 and an allele coverage filter based on a sequencing error rate of 0.01. The nQuire was subsequently used to calculate the deviation of every sample from the diploid, triploid and tetraploid models; therefore, the putative ploidy was indicated by the model with the lowest deviation. nQuack followed the same procedure while directly providing the membership likelihood for every ploidy class.

## 5. Conclusions

In conclusion, the ddRADseq approach employed in this study demonstrated its effectiveness in uniquely discriminating lantana clonal lines and in providing genetic parameters that characterize the germplasm collection of interest for breeding. Notably, this revealed the high degree of homozygosity present within the collection, leading to the consideration of the possibility for the species to present a predominant autogamous reproduction system. Furthermore, the molecular profiles derived from ddRADseq emerged as promising tools for the estimation of plant ploidy levels, avoiding the need for additional and not always feasible analyses, although further testing is needed to refine these predictions. Our findings suggest the advantages of the use of high-throughput sequencing techniques for genotyping and enhancing the technological advancement and precision of breeding programs. Additionally, this approach encourages the adoption of GBS techniques to generate molecular profiles that can support PVP and the fulfilment of PBRs. The integration of molecular and morphological descriptors may also be greatly beneficial for DUS testing. Ultimately, further research is needed to enhance our genetic and molecular understanding of minor species such as *L. camara*, for which many biological aspects remain to be elucidated. The ultimate goal of this study is to develop innovative varieties that are not only aesthetically appealing for their plant phenotypes to match the market demands but also sustainable and capable of providing valuable ecosystem services, thereby addressing the environmental and genetic challenges of the future.

## Figures and Tables

**Table 1:** Triangular genetic similarity (GS) matrix based on pairwise comparisons with the simple matching (SM) coefficient relative to 6 clusters obtained through an unweighted pair group method with arithmetic mean (UPGMA) clustering based on SNP profiles of 96 lantana breeding clonal lines.

Cluster	GS					
Cluster 1						
Cluster 2	89.85%					
Cluster 3	88.61%	91.35%				
Cluster 4	85.34%	85.69%	86.31%			
Cluster 5	88.14%	93.61%	91.20%	85.60%		
LaMo	55.15%	58.42%	56.45%	60.55%	62.39%	
	Cluster 1	Cluster 2	Cluster 3	Cluster 4	Cluster 5	LaMo

**Table 2:** Genetic statistics relative to 6 clusters obtained through the unweighted pair group method with arithmetic mean (UPGMA) clustering on the basis of SNP profiles of 96 Lantana breeding clonal lines. N: number of lines; Na: number of observed alleles per locus; Ne: number of effective alleles per locus; Ho (%): observed heterozygosity; He (%): expected heterozygosity; PA (%): percentage of private alleles among total population alleles; PL (%): percentage of polymorphic loci (%) among total population alleles.

Cluster	N	Na	Ne	Ho (%)	He (%)	Fis	PA (%)	PL (%)
1	33	1.14	1.12	14.34%	15.31%	0.06	4.91%	73.05%
2	18	1.15	1.06	14.79%	15.30%	0.03	1.68%	69.87%
3	7	1.13	1.08	13.35%	14.26%	0.06	2.66%	72.05%
4	25	1.19	1.15	18.99%	18.00%	-0.05	3.36%	70.06%
5	10	1.14	1.05	13.85%	14.20%	0.02	0.45%	62.47%
LaMo	3	1.07	1.51	28.29%	39.23%	0.28	11.27%	2.76%
Avg	16	1.14	1.16	17.27%	19.38%	0.07	4.05%	58.38%

**Table 3:** Results of genome size analysis through flow cytometry relative to the fluorescence intensity of the nuclei of two *Lantana camara* L. samples, with *Glycine max* Merr. cv. “Polanka” and *Pisum sativum* L. cv. “Ctirad” used as references [19].

Sample ID	Reference	Sample						Reference						Ref. Genome Size (pg)	Sample Genome Size (pg)	Avg Sample Genome Size (pg)
		Events	% of Vis	Mean	GeoMean	Median	CV	Events	% of Vis	Mean	GeoMean	Median	CV			
LaCaBL33-1	*G. max*	3273	27.3	10,480.4	10,470.1	10,500	4.4	11,233	53.1	5692.4	5683.4	5647	5.7	2.5	4.6	4.5
	*P. sativum*	3477	22.9	10,458.9	10,451.6	10,493	3.6	7080	44.7	21,771.6	21,762.6	21,791	2.9	9.1	4.4	
LaCaBL32-1	*G. max*	2173	14.1	12,588.6	12,585.2	12,602	2.3	7591	49.2	5022.2	5018.7	5021	3.7	2.5	6.3	6.1
	*P. sativum*	1821	23.3	13,023.8	13,015.1	13,080	3.6	2756	35.3	20,045.1	20,034.1	20,101	3.3	9.1	5.9	

**Table 4:** Results of ploidy analysis through flow cytometry relative to the fluorescence intensity of the nuclei of 15 *Lantana camara* L. samples, with the LaCaBL32-1 tetraploid accession used as a control. Tetraploid lines showed a single peak, due to the overlapping of sample and control signal.

Sample ID	Sample						Control (LaCaBL32-1)						Median Sample/ Control	Ploidy
	Events	% of Vis	Mean	GeoMean	Median	CV	Events	% of Vis	Mean	GeoMean	Median	CV		
LaCaBL06-3	2240.0	21.5	4221.8	4214.7	4217	5.8	1987.0	19.1	8365.9	8359.6	8368	3.9	2.0	Diploid
LaCaBL06-5	9917.0	21.3	4395.4	4381.2	4391	8.0	10,634.0	22.8	8621.7	8608.4	8611	5.6	2.0	Diploid
LaCaBL13-4	10,056.0	28.5	5693.3	5682.3	5695	6.2	3401.0	9.7	11,292.4	11,283.7	11,301	3.9	2.0	Diploid
LaCaBL23-1	6753.0	25.2	4601.4	4589.7	4601	7.1	3254.0	12.1	9147.1	9134.2	9155	5.3	2.0	Diploid
LaCaBL25-2	3612.0	20.8	4608.8	4597.4	4609	7.0	2277.0	13.1	8690.1	8682.6	8677	4.2	1.9	Diploid
LaCaBL11-1	4573.0	14.8	5230.2	5227.0	5236	3.5	7765.0	25.2	10,500.1	10,496.7	10,515	2.5	2.0	Diploid
LaCaBL01-1	3249.0	31.6	6762.2	6759.0	6760	3.1	1558.0	15.2	8877.2	8874.3	8881	2.6	1.3	Triploid
LaCaBL09-5	1357.0	14.5	5810.5	5807.3	5805	3.3	1439.0	15.4	7437.3	7434.4	7430	2.8	1.3	Triploid
LaCaBL010-1	748.0	21.5	8367.1	8363.8	8362	4.3	2125.0	21.6	10,863.9	10,861.1	10,871	2.3	1.3	Triploid
LaCaBL33-1	642.0	31.3	7311.4	7307.5	7309	3.2	277.0	13.5	9882.7	9880.9	9884	1.9	1.4	Triploid
LaCaBL05-1	2793.0	15.0	11,179.0	11,172.4	11,188	3.4	-	-	-	-	-	-	-	Tetraploid
LaCaBL31-1	1355.0	48.1	12,151.0	12,145.8	12,157	2.9	-	-	-	-	-	-	-	Tetraploid
LaCaBL32-1	1548.0	40.7	10,373.4	10,368.8	10,372	3.0	-	-	-	-	-	-	-	Tetraploid
LaCaBL34-1	4089.0	41.0	11,885.2	11,880.9	11,876	2.7	-	-	-	-	-	-	-	Tetraploid
LaCaBL35-1	866.0	49.6	11,795.9	11,790.4	11,774	3.1	-	-	-	-	-	-	-	Tetraploid

## Data Availability

ddRAD-seq data presented in the study are openly available in GenBank under the BioProject accession number PRJNA1257841.

## Supplementary Materials

The following supporting information can be downloaded at: https://www.mdpi.com/article/10.3390/ijms26104898/s1.
